# Investigating the impact of storage duration and temperature on vitamin C in various citrus genotypes: A meta-analysis method

**DOI:** 10.1016/j.mex.2024.102742

**Published:** 2024-05-03

**Authors:** Rahmat Budiarto, Danung Nur Adli, Teguh Wahyono, Tri Ujilestari, Mohammad Miftakhus Sholikin, Syariful Mubarok, Dwi Novanda Sari, Ana Khalisha, Stefina Liana Sari, Supatida Abdullakasim

**Affiliations:** a Department of Agronomy, Faculty of Agriculture, Universitas Padjadjaran, Sumedang 45363, Indonesia; b Meta-Analysis in Plant Science (MAPS) Research Group, Bandung 40621, Indonesia; c Feed and Animal Nutrition Department, Faculty of Animal Science, Universitas Brawijaya, Malang 65145, Indonesia; d Research Center for Food Technology and Processing, National Research and Innovation Agency (BRIN), Gunungkidul, 55861, Indonesia; e Research Center for Animal Husbandry, Research Organization of Agriculture and Food, National Research and Innovation Agency (BRIN), Bogor 16915, Indonesia; f Study Program of Agrotechnopreneur, Faculty of Agriculture, Universitas Padjadjaran, Sumedang 45363, Indonesia; g Department of Horticulture, Faculty of Agriculture at Kamphaeng Saen, Kasetsart University, Kamphaeng Saen Campus, Nakhon Pathom, Thailand

**Keywords:** Genetic factor, Meta-analysis method, Scientific protocol, Storage practices, Topic selection, A meta-analysis method to consolidate the impact of storage duration and temperature on vitamin C in various citrus genotypes

## Abstract

The present work disseminates a solid scientific meta-analysis method to investigate the impact of storage duration and temperature on vitamin C of citrus. This work is initiated by designing of the PICO framework, collecting, and organizing the articles, creating selection criteria, sorting articles, identifying factors influencing moderation effects and sources of diversity, tabulating data, and employing analysis in the form of a linear mixed model. Using this method, we identified 54 distinct studies from a pool of 289 eligible peer-reviewed publications, focusing on variations of vitamin C in citrus. The method provides mean values in both quadratic and linear regression forms.

• This method provides a detailed description starting from topic selection to statistical methodologies intended for performing meta-analysis.
• All guidelines for conducting this method have been approved by all authors and adhere to the standard PRISMA-P guidelines.
• Disseminating this method in a peer-reviewed publication aims to facilitate scholarly discussions and promote transparency, ultimately improving the standard for performing meta-analysis on vitamin C levels in citrus concerning various genotypes, storage temperatures, and durations.

## Graphical abstract

**Figure:**
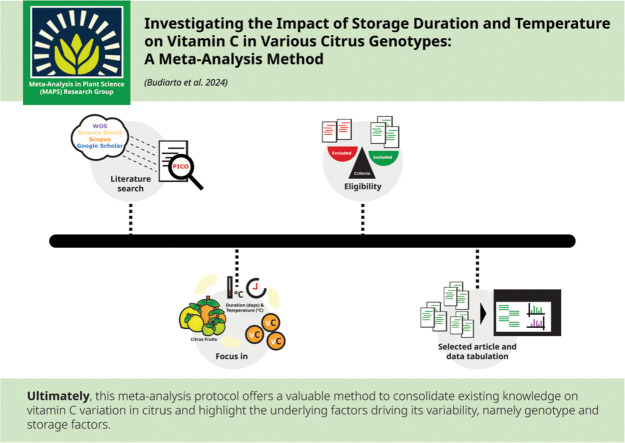

Specifications table

**Table:** 

Subject area:	Agricultural and Biological Sciences
More specific subject area:	Post-Harvest Management
Name of your method:	A meta-analysis method to consolidate the impact of storage duration and temperature on vitamin C in various citrus genotypes
Name and reference of original method:	Not applicable
Resource availability:	Mendeley Desktop version 1.19.8, WebPlotDigitizer version 4.5, Microsoft Excel 2019 and R version 4.3.0

## Method details

### The goal of the meta-analysis method

We aim to conduct the meta-analysis examining the variation in vitamin C content based on citrus genotypes and storage conditions, adhering to stringent meta-analytical quality criteria, including selection and dataset development. The research questions are formulated using the PICO framework [1,2], which focuses on the population (P) of experimental units, intervention (I) or treatment, comparison (C) against control, and outcome (O). The meta-analysis separates the factors of storage duration and temperature in relation to the vitamin C content of citrus fruits (Table 1). Variability that may affect data bias, such as citrus fruit varieties, is minimized by conducting a breakdown at the data analysis stage. The formulation of research questions following the PICO framework is as follows:

A. Storage Duration Factor
  a. *P* = How does the duration of storage affect the vitamin C content of citrus fruits?
  b. *I* = What is the effect of changes in storage duration on vitamin C concentration?
  c. *C* = How does storage condition on day 0 (post-harvest) compare to longer storage durations in terms of citrus fruit vitamin C concentration?
  d. *O* = What is the vitamin C concentration after exposure to extended storage duration?
B. Storage Temperature Factor
  a. *P* = How do changes in citrus fruit storage temperature affect its vitamin C concentration?
  b. *I* = What changes in vitamin C result from changes in storage temperature?
  c. *C* = How does the treatment comparison between storage at 0 °C and higher temperatures affect the vitamin C content of citrus fruits?
  d. *O* = What is the vitamin C content of citrus fruits after exposure to temperature storage treatment?

**Table 1:** Requirements for eligibility.

PICO	Description
Storage duration	
*Population*	Citrus fruits
*Intervention*	Length of storage
*Comparator*	Storage duration of 0 days as control
*Outcome*	Changes in vitamin C levels in citrus fruits
Storage temperature	
*Population*	Citrus fruits
*Intervention*	Storage temperature
*Comparator*	Temperature of 0 °C as control
*Outcome*	Changes in vitamin C levels in citrus fruits

The design of the above questions is then translated into the PICO framework and the construction of keyword combinations for secondary literature search (in methodology section) [3,4]. The main limitations of our study include the highly diverse nature of citrus fruits in terms of varieties and climatic factors such as elevation, duration of light exposure, storage humidity, oxygen, and nitrogen density, environmental cleanliness during research/storage, and other environmental factors. These diverse factors are challenging to trace both in the methodology section and the availability of records in the results section. Therefore, these factors are excluded, and based on the experimental design used, whether it is a completely randomized design, a randomized block design, or a factorial design from the environmental design only accommodates differentiating environmental factors (treatments), while other factors are considered homogeneous or homogenized. Taking into consideration other environmental factors alongside storage duration and temperature, which are assumed to remain constant, further examination is conducted on the genotype variations of citrus varieties, which determine the variability in vitamin C. Storage duration ranges from 0 to 180 days, and recorded storage temperatures range from 0 to 40 °C, detailed in the data description section. Identified citrus fruit types include Grapefruit (*Citrus paradisi*), Hybrid (unspecified hybrid type), Lemon (*Citrus limon*), Lime (*Citrus aurantiifolia* and *Citrus latifolia*), Mandarin (*Citrus reticulata*), Orange (*Citrus sinensis* and *Citrus aurantium*), and Tangerine (*Citrus reticulata* var. tangerine).

### Collecting and organizing the articles

The keywords were organized based on Table 2, in accordance with the modified PICO framework [5,6]. The search was conducted using the Web of Science, Science Direct, Scopus, and Google Scholar search engines. Further details regarding the variations in keyword usage are explained in Annex 1. The main components that needed to be included in the search were the keywords “citrus” or “orange,” “vitamin C” or “ascorbic acid,” and “storage duration” or “storage temperature”. Literature sources such as student theses and dissertations were not considered in the search. Primary research from experimental designs published in journals and indexed with digital object identifiers (DOIs) in English was deemed the ideal reference source for this meta-analysis. While data sources related to the topic can also be found in gray literature and scientific repositories from universities, these reference sources were not considered reliable as the data from such literature have not undergone the peer-review system. Therefore, the use of DOIs selectors can exclude non-peer-reviewed articles as a source.

**Table 2:** Keywords arranged for tracing references in meta-analysis. Adapted from Budiarto and Sholikin (2022) [6].

Population		Intervention		Output
Storage duration				
(citrus* OR orange*)	AND	(“storage duration”)	AND	(“vitamin C” OR “ascorbic acid”)
Storage temperature				
(citrus* OR orange*)	AND	(“storage temperature”)	AND	(“vitamin C” OR “ascorbic acid”)

There were no restrictions on the search date, and limitations were the default settings of the search engine system. Similarly, the search scope was not restricted. Limitations were only imposed on reference sources originating from experimental research and journals indexed with digital object identifiers (DOIs) and written in English.

The search results in *.ris file format was imported into Mendeley Desktop version 1.19.8. The application automatically identified duplicated data, and manual verification was conducted for duplicate data. Once it was confirmed that the articles were identical, a merging process was undertaken. The initial relevance assessment of articles included in this meta-analysis was conducted in the early stage using the reference of titles and abstracts (Table 3). If the article met the criteria outlined in the previously provided PICO framework, it moved on to the next step for the overall selection process of the article content. Important notes regarding the search process and tabulation of reference sources can be found in the annex (Annex 1). Possible synonyms for each of the words above include citrus* or orange* (citrus fruits, lemon, lime, grapefruit, tangerine, and mandarin), storage duration (shelf life, expiration period, and keeping time), storage temperature (ambient temperature and climate control), and vitamin C or ascorbic acid (L-ascorbic acid).

**Table 3:** Selection criteria for each section (title, abstract, method, and results).

	Inclusion criteria	Exclusion criteria
**1.**	**General**	
1.a.	The article is written in English.	The article uses a language other than English.
1.b.	Having digital object identifiers (DOIs).	Do not have DOIs.

**2.**	**Title**	
2.a.	*P* = Must specify the variety or type of citrus fruit that contains vitamin C or its equivalent.	*P* = Other subjects, besides citrus fruits or their equivalents, containing vitamin C and/or equivalent.
2.b.	*I* = There is a treatment phrase such as storage period or storage temperature, or their equivalents.	*I* = Treatment related to the duration of storage and storage temperature is not mentioned in the title.
2.c.	*O* = Implicitly mention that measurements of vitamin C content, ascorbic acid, or similar substances are conducted.	*O* = Does not explicitly state the test parameter results regarding the vitamin C content or ascorbic acid or qualitative vitamin C testing results and does not provide continuous numeric data.

**3.**	**Abstract**	
3.a.	*P* = The abstract section explains the type of citrus used, as well as any information regarding their origin and harvest time.	*P* = The type of citrus used is not mentioned and is difficult to identify using previous research conducted by the same researcher or research group.
3.b.	*I* = Clearly stating the storage duration or temperature of citrus as factors affecting vitamin C levels.	*I* = There is no data or statement regarding the storage duration or temperature of citrus and their influence on vitamin C.
3.c.	*C* = Presence of treatment control.	*C* = Absence of treatment control.
3.d.	*O* = Mentioning the measurement of vitamin C levels, including methods and results.	*O* = There is no information provided regarding the vitamin C values, methods, or results.

**4.**	**Method**	
4.a.	*P* = Explaining information about the process of handling citrus before storage (treatment conditions) and when measuring their vitamin C levels.	*P* = There is no clear treatment mentioned, and environmental treatment is not specified with certainty.
4.b.	*I* = Mentioning and explaining the vulnerability of long storage treatment or temperature storage vulnerability.	*I* = The vulnerability of storage temperature and/or storage duration is unknown.
4.c.	*C* = Explicitly describing treatments that compare with the control, which is the treatment for 0 days of storage duration and 0 °C storage temperature.	*C* = Absence of control in treatments, both for storage duration and storage temperature.
4.d.	The types of testing methods for vitamin C referred to are iodometric method, titrimetric method, spectrophotometric method, chromatography, biological methods, or similar ones, aiming to quantify the level of vitamin C.	Other unverified methods for testing vitamin C that have difficulty demonstrating the value of vitamin C levels.

**5.**	**Output**	
5.a.	*O* = The level of vitamin C or ascorbic acid and similar substances.	*O* = Failure to include data on vitamin C or ascorbic acid and similar substances; furthermore, if the data is qualitative and does not reflect standardized quantitative values, it cannot be used.
5.b.	The unit of vitamin C levels is expressed in mg/100 g of fresh weight or similar units.	Failure to include units or present the data in percentage form, along with unknown total standard concentrations, complicates the recalculation process.
5.c.	The type of data used is the average measurement of vitamin C.	The type of data consists of qualitative measurements of vitamin C and/or data presented as relative percentages.

^1^*C* = comparison between control *vs*. treatment (storage duration and temperature), *I* = intervention or treatment given, which is storage duration and temperature treatment, *P* = population of the study or the topic of this meta-analysis research, which is oranges, *O* = refers to the results obtained from each experimental study from secondary sources in terms of vitamin C content in mg/100 g of fresh citrus.

### Creating selection criteria and sorting of articles

The literature obtained during the search phase will undergo further selection using the criteria outlined in Table 3. These criteria include both inclusion and exclusion criteria based on the PICO framework. Checking will be performed using specific criteria (Table 3) applied to each section of the title, abstract, methods, and results.

**Fig. 1:**
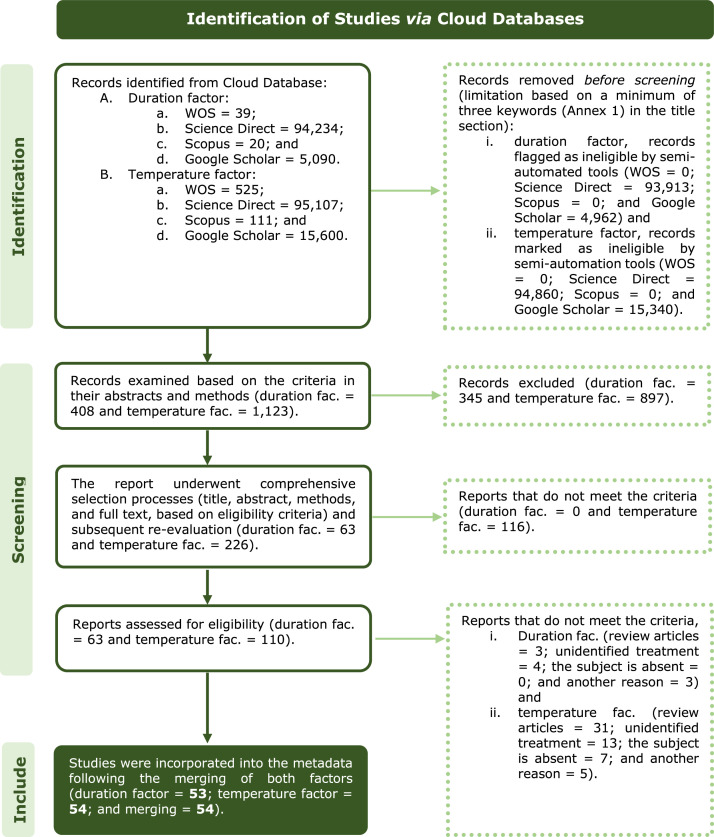
Prisma flow diagram of literature retrieval [7,8].

If the article meets the inclusion criteria numbers 1 through 5, then it qualifies for consideration as one of the data sources for this meta-analysis, or the article has met the criteria in every aspect of its title, abstract, methods, and results. To ensure the validity of the data obtained from the article sources, a specific assessment regarding data reliability is conducted, with the range values for ascorbic acid or vitamin C in citrus used as the reference point. The normal values for vitamin C in citrus range from 30 to 70 mg/100 g of citrus, across various species. Additionally, to ensure the selection process, a two-stage selection is carried out with four individuals as selectors and two individuals as validators. The selection process, which employs the selection criteria in Table 2, is further detailed in the flowchart shown in Fig. 1.

### Factors influencing moderation effects and sources of diversity

Moderation effects on the treatment of storage influence (duration and temperature) on the vitamin C content of citrus can be explained by various variables that directly or indirectly affect both relationships between clauses. Further explanations regarding the moderator effect variables can be found in Table 4. Additionally, the sources of diversity, which contribute to the variation in data derived from various studies, are described in the context of descriptive clauses, including variables and their respective groups or ranges as shown in Table 4. The moderator effect elucidates the independent variables that can potentially bring about significant changes in the vitamin C content of citrus fruits. For instance, planting systems, such as organic or intensive systems, clearly exhibit significant differences. Additionally, the randomization of research regarding the vitamin C content in citrus due to variations in temperature and storage duration can reduce the level of error arising from the moderator effect. Moreover, these moderator factors do not have a direct effect that could intervene in the running of postharvest-based research.

**Table 4:** Moderation effects and diversity sources of long-term treatment and storage temperature on citrus fruit vitamin C levels.

	Explanation and explanation groups
**Moderation effect**	
The harvesting age of citrus	The citrus used are either freshly harvested or up to a maximum of less than one day post-harvest.
Treatment	
Storage duration (days)	The storage duration is not restricted; however, the measurement of vitamin C levels is taken at 0 days of storage.
Storage temperature (°C)	The range of storage temperatures is not restricted.
Characteristics of citrus	
Types of citrus (varieties)	There is no limit to the varieties of citrus, but at least more than one experiment discusses the changes in vitamin C content of these varieties.
Planting	
Planting system	Conventional farming system and organic farming system, factors related to planting distance, harvesting handling system, and the utilization of pesticides or herbicides.
Types of fertilizers used	Organic and synthetic fertilizers
Climatic and geolocation influences	Rainfall, altitude, humidity, and temperature.
Measurement methods for vitamin C	Different methods for measuring vitamin C, including basic methods and their developments. These include titrimetric-based, spectrophotometry, electrochemistry, enzyme-based methods, high-performance liquid chromatography, and biosensors.
**Source of diversity**	
Experimental design	Environmental design that allows for complete random design, group random design, and factorial design, whereas other designs like split-plot based designs cannot be used because the type or variety of citrus fruit is not included in existing species so it is definitely sufficient to use the above designs.
Population characteristics (sample or trial populations in testing)	A minimum of three trials may be used if *n* = 3.
Research bias	The globally agreed bias is an error bias within a range of 1 % or 5 % from the variation testing (ANOVA).

### Data tabulation procedure and analysis

Data collected from 54 selected studies consist of average values from treatment and control groups. Extraction of data from image sources was carried out using WebPlotDigitizer version 4.5, employing the technique of comparing distances on the X’ and Y’ axis projections with actual X or Y axis values. The gathered data was tabulated in Microsoft Excel 2019, resulting in 1436 rows of raw data. Essential column information includes: study number, citrus fruit species, citrus fruit group, storage duration (days), storage temperature ( °C), and vitamin C content (in mg/100 g fresh weight). The database documents the source and type of citrus fruits as presented in Table 5. Finally, 34 data points were discarded due to non-measurable vitamin C values.

**Table 5:** Origin and types of citrus fruits recorded in the database.

	Note
**Citrus origin**	Africa
	Egypt: Ismailia
	Morocco: Rabat
	Sudan: Khartoum
	Tanzania: Tanga
	America
	US: California, Lake placid, South Texas, and Texas
	Australia
	Neergabby
	Queensland
	Asia
	China; Ganzhou, Guangdong, Hangzhou, Xiangtan, and Zhejiang
	India: Jabalpur, Nagpur, and Punjab
	Iran: Darab and Khuzestan
	Israel: Hachula valley and South coast
	Pakistan: Punjab, Peshawar, Sargodha, and Swat
	South Korea: Daegu
	Taiwan: Taichung and Yilan Orchard
	Europe
	Italy; Sardinia, Siracusa, and Oristano
	Poland; Poznan
	Spain; Valencia
	Turkey: Antalya, Hatay, and Mersin
	South America
	Argentine: Corrientes
	Brazil: Araraquara, Amambai, Jaguaribe, and Porto Alegre
**Citrus species**	Afourer mandarins
	Champagne orange
	Clemenules mandarin
	Daegu mandarin
	Delta valencia orange
	Duncan grapefruit
	Eureka lemon
	Fortune
	Kagzi acid lime
	Kagzi lime
	Key acid lime
	Kinnow mandarin
	Minneola tangelos(hybrid)
	Montenegrina and Rainha tangerines
	Moroccan mandarin
	Mosambi orange
	Msasa and Jaffa oranges
	Murcott tangor (hybrid)
	Mutant mandarins (Prenules, Basol, Clemenrubí, and Orogros)
	Nadorcott mandarin
	Nagpur mandarin
	Navelina oranges
	Nova
	Oronules mandarin
	Pakistani blood red oranges
	Ponkan mandarin
	Rio red grapefruit
	Sanguinello and Doppio
	Satsuma mandarin
	Siavarz orange
	Sinnari green oranges
	Star ruby grapefruit
	Tankan mandarin
	Torocco and Moro oranges
	Turkish mandarin
	Valencia oranges

The data analysis of the meta-analysis follows the framework proposed by Sauvant et al. [8] and St-Pierre [9], employing a linear mixed model (LMM). In this model, the treatment of vitamin C is considered as the fixed effect, while the between-study error is accounted for as a random effect. The mathematical model adopted is a modification of the one presented by Sholikin et al. [10].

(1) $$Y_{ij}\;=\;\mu \;+\;s_{i}\;+\;\tau_{j}\;+\;s\tau_{ij}\;+\;\beta_{0}+\;\beta_{1}S_{ij}+\;b_{i}S_{ij}+\beta_{2}{S_{ij}}^{2}+\;b_{i}{S_{ij}}^{2}+\;e_{ij}$$

Notes: $Y_{ij}$ - represents a dependent variable, μ - denotes the overall mean value, $s_{i}$ - signifies the random effect from the *i*^−th^ study, under an assumption of being normally distributed with mean 0 and variance $∼\;N_{iid}\;\left(0,\;\sigma_{S}^{2}\right)$, $\tau_{j}$ - indicates the fixed effect associated with the *j*th level of the τ factor, $s\tau_{ij}$ - represents a random interaction between the *i*th study and the *j*th level of the τ factor, assuming a normal distribution with mean 0 and variance $∼\;N_{iid}\;\left(0,\;\sigma_{S\tau }^{2}\right)$, $\beta_{0}$ -stands for the intercept value representing the average of all studies intersecting the axis, $\beta_{1}$ -serves as the regression coefficient, $S_{ij}$ and ${S_{ij}}^{2}$ - denote the linear and quadratic predictors, specifically the storage period (in days) and storage temperature (in °C), $b_{i}$ - represents the random effect accounting for differences among studies in the regression coefficient Y relative to the predictor X in the *i*th study, and $e_{ij}$ represents the unexplained error value.

Statistical validation was conducted using the root mean square error (RMSE) and determination coefficient (R^2^) in R version 4.3.0 [[11], [12], [13]], according to the following equations.

(2) $$RMSE\;=\;\sqrt{\frac{\sum {\left(A-E\right)}^{2}}{n}}$$

(3) $$R^{2}=\;\frac{\left({s^{2}}_{f}\;+\;\sum \left({s^{2}}_{l}\right)\right)}{\left({s^{2}}_{f}\;+\;\sum \left({s^{2}}_{l}\right)+\;{s^{2}}_{e}+\;{s^{2}}_{d}\right)}$$

In this equation, A - represents actual data, E - stands for estimated data, n - denotes the total number of data points, ${s^{2}}_{f}$ - refers to the variance attributed to a fixed factor, $\sum ({s^{2}}_{l})\;$- represents the sum of all component variances. Additionally, ${s^{2}}_{e}$ - accounts for the variance caused by predictor dispersion and ${s^{2}}_{d}$ - denotes the specific distribution variance.

### Description of the dataset used in this meta-analysis

We gathered numerous data points from 54 individual studies on citrus vitamin C, which varied according to origin, genotype, storage duration, and temperature. Initially, in our meta-analysis, the dataset compiled included the number of data points concerning vitamin C in the current meta-study, reporting both the mean and maximum values. Storage duration, storage temperature, and citrus species were also considered. Secondly, our results presented the number of data points (NDP), with reported values and standard errors (SE) for both the intercept and slope. Additionally, validation results included the root mean square error (RMSE), and the p-value. Discrepancies often arose among the various individual studies during the initial stage of dataset construction for the meta-analysis. The interaction among different storage durations, species, and temperatures was also noted to help reduce bias.

### Method and implementation implications

The implications of the referencing method in examining the effects of storage temperature, duration, and genotype variation on vitamin C content are that, through rigorous meta-analysis and selective criteria, this method can provide a precise understanding of how these three factors can influence the vitamin C content of citrus fruits. This is beneficial for post-harvest handling to enhance the quality and shelf life of citrus.

The future implementation related to this formulated method can serve as a reference framework for the preparation of topic-based manuscripts on post-harvest technology, supported by the use of appropriate scientific frameworks such as the application of PICO in idea formulation procedures, secondary article searches, and weighted literature selection processes. It particularly minimizes limitations related to external factors other than temperature and storage duration, which are complex, as well as variations in citrus fruits.

## Method validation

### Annex

#### Recording the findings of a literature search

A. **Duration factor**
  a. **Web of Science**

  **Search database:** indexing is carried out on the following database,

• Science Citation Index Expanded (SCI-EXPANDED) from 1970 to present,
• Conference Proceedings Citation Index – Science (CPCI-S) from 1990 to present,
• Book Citation Index – Science (BKCI-S) from 2005 to present, and
• Emerging Sources Citation Index (ESCI) from 2005 to present.

**Field:** in the study, searches were conducted by indexing titles, abstracts, and keywords from literature, referred to as TS (topic search). Additionally, ALL (searching all available fields) was utilized to enable the convenient location of search terms within any field for a comprehensive search.
**Search period:** default (1970–2024) and result (2007–2023).
**Search keyword query:** TS = (citrus* OR orange*) AND TS = (vitamin C OR ascorbic acid) AND ALL = (storage duration) AND ALL= (storage*).
**Number of literatures:** article (38) and proceeding paper (1).

  b. **Science Direct**

**Search database:** no additional details were accessible at the time.
**Field:** the specification regarding the indexing methodology of terms was not provided.
**Search period:** result (2001–2025).
**Search keyword query:** citrus OR orange AND “vitamin C” OR “ascorbic acid” AND “storage duration”.
**Number of literatures:** review articles (10,399), research articles (56,217), encyclopedia entries (1662), book chapters (13,231), conference abstracts (1602), book reviews (425), case reports (109), conference information (100), correspondence (224), data articles (60), discussion pieces (416), editorials (271), errata (61), examinations (11), mini reviews (507), news articles (226), patent reports (51), practice guidelines (33), product reviews (40), short communications (2276), software publications (2), and other types of literature (4921).
**Limitation:** because of Science Direct unreliable search accuracy, we organized the titles of articles that fulfilled our criteria. These criteria mandated a minimum presence of factors like (citrus OR orange) AND (vitamin C OR ascorbic acid) AND (storage duration). Consequently, we identified a total of 321 articles conforming to our experimental design.

  c. **Scopus**

**Search database:** literature published in SCOPUS.

**Field:** TITLE-ABS-KEY (searching requires the indexing of titles, abstracts, and keywords from literature).

**Search period:** default (under 1960 to present) and result (2007–2023).

**Search keyword query:** (TITLE-ABS-KEY (citrus* OR orange*) AND TITLE-ABS-KEY (“vitamin C” OR “ascorbic acid”) AND TITLE-ABS-KEY (“storage duration”) AND TITLE-ABS-KEY (storage*)).

**Number of literatures:** journal (18), book series (1), and conference proceeding (1).

  d. **Google Scholar**

**Search database:** further details concerning the origin of the database remained undisclosed.

**Field:** no specific details were ascertainable.

**Search period:** without imposing any limitations on publication years.

**Search keyword query:** citrus OR orange AND “vitamin C” OR “ascorbic acid” AND “storage duration”.

**Number of literatures:** the overall count of articles captured (5090) lacking further detailed categorization was noted.

**Limitation:** due to the inconsistent search accuracy of Google Scholar, we categorized the titles of articles that met our criteria. This required including at least (citrus OR orange) AND (vitamin C OR ascorbic acid) AND (storage duration). This resulted in a total of 128 articles that were selected based on their experimental design.

B. **Temperature factor**
  a. **Web of Science**

**Search database:** indexing was executed on the subsequent database,

• Science Citation Index Expanded (SCI-EXPANDED) from 1970 to present,
• Conference Proceedings Citation Index – Science (CPCI-S) from 1990 to present,
• Book Citation Index – Science (BKCI-S) from 2005 to present, and
• Emerging Sources Citation Index (ESCI) from 2005 to present.

**Field:** the search was carried out by indexing titles, abstracts, and keywords from literature for the TS (Topic Search) and ALL (All Fields) options. This allowed for a comprehensive search, facilitating the convenient identification of search terms within any field.

**Search period:** default (1970–2024) and results (1977–2024).

**Search keyword query:** TS = (citrus* OR orange*) AND TS = (vitamin C OR ascorbic acid) AND ALL = (storage temperature) AND ALL = (storage*).

**Number of literatures:** article (464), proceeding paper (35), review (17), early access (5), book chapters (2), meeting abstract (1), and note (1).

  b. **Science Direct**

**Search database:** no further information was available at the time.

**Field:** the indexing process failed to furnish specifics regarding the methodology employed for terms.

**Search period:** result (2001–2025).

**Search keyword query:** citrus OR orange AND “vitamin C” OR “ascorbic acid” AND “storage temperature”.

**Number of literatures:** review articles (10,674), research articles (57,446), encyclopedia (1732), book chapters (13,673), conference abstracts (1644), book reviews (425), case reports (110), conference info (100), correspondence (225), data articles (60), discussion (421), editorials (271), errata (61), examinations (11), mini reviews (510), news (227), patent reports (59), practice guidelines (33), product reviews (40), short communications (2301), software publications (2), and other (5010).

**Limitation:** as a result of Science Direct unreliable search accuracy, we sifted through article titles that fit our criteria, necessitating the presence of at least one of the following: “citrus” or “orange,” combined with “vitamin C” or “ascorbic acid,” and “storage temperature.” This process yielded a sum of 247 articles with experimental designs.

  c. **Scopus**

**Search database:** publications indexed in SCOPUS.

**Field:** TITLE-ABS-KEY (searching involves indexing literature titles, abstracts, and keywords).

**Search period:** default (under 1960 to present) and result (1948–2024).

**Search keyword query:** (TITLE-ABS-KEY (citrus* OR orange*) AND TITLE-ABS-KEY (“vitamin C” OR “ascorbic acid”) AND TITLE-ABS-KEY (“storage temperature”) AND TITLE-ABS-KEY (storage*)).

**Number of literatures:** article (98), conference paper (11), and review (2).

  d. **Google Scholar**

**Search database:** we lacked further information regarding the database's origins.

**Field:** there is no particular information accessible.

**Search period:** without any limitations on the year of publication.

**Search keyword query:** citrus OR orange AND “vitamin C” OR “ascorbic acid” AND “storage duration”.

**Number of literatures:** the overall count of seized items (15,600) without further detailed classification.

**Limitation**: because of Google Scholar unreliable search precision, we screened article titles based on our criteria, necessitating the presence of at least one of the following elements: (citrus OR orange) AND (vitamin C OR ascorbic acid) AND (storage temperature). This process yielded a sum of 260 articles meeting our experimental design requirements.

*Literature tabulation of search results for both factors (duration and storage temperature) and removal of duplicate sources.*

A. **Mendeley:**

The utilization of Mendeley Desktop version 1.19.8 for managing literature acquired from searches on cloud databases was undertaken. Subsequently, each search result, categorized by storage duration and storage temperature, was segregated into different storage directories. Publication duplicates were automatically traced and manually verified to ensure the validity of duplicate articles. Finally, the duplicated articles underwent a merging process to consolidate them into a single article.

B. **Manual checking:**

The articles identified as duplicates by the Mendeley system were subjected to manual validation, wherein data was sorted according to publication titles. When two identical titles were found, a merging process was initiated.

## Additional information

### The determination of the issue

Before becoming a globally popular commodity, citrus was believed to have originated in specific Asian regions [[14], [15], [16]]. The primary factor driving the popularity of citrus is its delicious and nutritious fruit, as highlighted by Nicolosi [17], Wang et al. [18], which contains a variety of beneficial phytochemicals [[19], [20], [21]]. The vitamin C content is influenced significantly by both genetic factors, as discussed by Escobedo-Avellaneda et al. [22], Magwaza et al. [23], Sdiri et al. [24], and environmental conditions, as emphasized by Fotopoulos et al. [25]. Various genotypes may exhibit different vitamin C contents, as reported by numerous researchers at both inter- and intra-species levels [[26], [27], [28]]. Environmental factors, such as the plant growing location, lead to variations in climatic, edaphic, and applied cultural practices, resulting in different vitamin C contents. Post-harvest modifications, particularly storage, are crucial for maintaining vitamin C content until it reaches the consumer. Prolonged storage times are associated with higher vitamin C loss [29,30].

Currently, there is limited information on studying Vitamin C Variation in Citrus in response to Genotypes, Storage Temperatures, and Periods. Rozane et al. [31] reported the selection of groups in citrus varieties but did not establish correlations among them. Tahir et al. [32] investigated the effect of the combination of bioactive compounds and antagonistic yeasts on postharvest diseases in fruits (*in vivo*) and estimated overall disease incidence on citrus. Our objective is to conduct the first meta-analysis on the variation of vitamin C based on citrus genotype and storage. We will adhere to meta-analytical quality criteria, including selection, dataset development, and bioinformatics computation. Through this approach, we aim to provide high-quality and novel insights into vitamin C variation based on genotype and storage.

## Ethics statements

The drafting of this manuscript has adhered to the writing standards permitted by MethodsX. The meta-analysis protocol regarding the topic of the effects of storage duration and temperature on vitamin C levels did not involve *in vitro* testing using cells, *in vivo* experimentation using laboratory animals (mice and/or other animals), or research involving human subjects. Additionally, the data used originated from recognized scientific repositories, and the necessary permissions related to the data have been fulfilled by citing the appropriate sources in this manuscript.

## CRediT authorship contribution statement

**Rahmat Budiarto:** Conceptualization, Funding acquisition, Methodology, Writing – review & editing. **Danung Nur Adli:** Supervision, Validation, Writing – original draft, Writing – review & editing. **Teguh Wahyono:** Validation, Software, Writing – original draft, Writing – review & editing. **Tri Ujilestari:** Project administration, Data curation, Writing – original draft. **Mohammad Miftakhus Sholikin:** Supervision, Validation, Visualization, Writing – review & editing. **Syariful Mubarok:** Conceptualization, Writing – original draft. **Dwi Novanda Sari:** Conceptualization, Writing – original draft. **Ana Khalisha:** Conceptualization, Writing – original draft. **Stefina Liana Sari:** Conceptualization, Writing – original draft. **Supatida Abdullakasim:** Writing – review & editing.

## Declaration of Competing Interest

The authors declare that they have no known competing financial interests or personal relationships that could have appeared to influence the work reported in this paper.

## Data Availability

No data was used for the research described in the article.
